# A cross-sectional study of Chinese women facial skin status with environmental factors and individual lifestyles

**DOI:** 10.1038/s41598-022-23001-6

**Published:** 2022-10-27

**Authors:** Fan Yi, Xiao-xiao Yang, Ru-ya Yang, Meng-meng Zhao, Yin-mao Dong, Li Li, Yi-fan He, Miao-miao Guo, Jing Li, Xiao-hui Zhang, Zhi Lu, Jie Gu, Jing-lin Bao, Hong Meng

**Affiliations:** 1 Key Laboratory of Cosmetic, China National Light Industry, Beijing Technology and Business University, Beijing, People’s Republic of China; 2 Eviskin Cosmetics Technology (Beijing) Co., Ltd., Beijing, People’s Republic of China; 3 Shanghai Inoherb Cosmetic Co., Ltd., Shanghai, People’s Republic of China

**Keywords:** Environmental social sciences, Health care, Public health, Skin diseases

## Abstract

Geographical, environmental and pollution conditions affect facial skin health, but their effects on skin appearance have not been elucidated. This study aimed to describe the skin barrier and skin tone characteristics of Chinese subjects according to lifestyle and environmental conditions using in vitro measurements. In total, 1092 women aged 22–42 years were recruited from 7 representative Chinese cities. Eight skin parameters (hydration, sebum, pH, transdermal water loss, individual type angle, melanin index, erythema index, yellowness) were measured using noninvasive instruments; individual lifestyle data were also collected. Data on four meteorological factors (air temperature, relative humidity, sunshine duration, wind speed) and seven air pollution indicators (air quality index, fine particulate matter, breathable particulate matter, sulfur dioxide, nitrogen dioxide, carbon monoxide and ozone) were collected in each city from the China Meteorological Administration. Facial skin characteristics differed significantly between cities. Facial skin barrier characteristics and skin tones showed regional differences, with a better skin barrier associated with the western region, as indicated by high skin hydration and sebum secretion and a low pH value. According to the value of transdermal water loss, lighter and darker skin tones were found in the western and southern regions, respectively. Environmental conditions affected facial skin status. Air pollution induced facial skin issues, with fine particulate matter and nitrogen dioxide contributing the most. Individual lifestyles affected the facial skin barrier and skin tone.

## Introduction

Facial skin status can reflect the skin characteristics of an individual and reveal health status^1–3^. In recent years, the development of non-invasive skin assessment instruments has made it possible to assess the skin status of volunteers in vitro. “Exposome” characterizes the total exposure of a person from conception to death. The key elements of the exposome influencing skin aging have been determined by epidemiological, in vitro, ex vivo and clinical studies^4^. However, the exposure effects of the facial skin barrier and skin tone have not received major attention, and a large-scale cross-sectional study of the exposome effect in Chinese populations is lacking. In this study, we evaluated the skin barrier and tone of Chinese women using a non-invasive instruments, and eight skin parameters (hydration, sebum, pH, transdermal water loss, individual type angle, melanin index, erythema index, yellowness) were measured (Table 1). Evidence for the effect of exposure on skin status in Chinese populations was provided through this cross-sectional study.

**Table 1:** Abbreviation, meaning and measurement equipment/sources of the parameters.

Abbreviation	Parameter meaning	Measure equipment and specifications
**Sect. 1 Abbreviation, meaning and measurement equipment of skin parameters**		
CM (a.u.)	Skin moisture content, a higher CM value indicates better skin hydration	Corneometer CM 825
TEWL (g/m^2^/h)	Transdermal water loss content,	Tewameter TM300
SM (μg/cm^2^)	Sebum secretion content, too high or too low SM value affects the normal barrier function of epidermis and lead to skin problems	Sebumeter SM815
pH	Skin pH, normal skin has a pH of 4.5 to 6.5	Skin-pH-Meter PH905
MEXA (a.u.)	Melanin content, a larger MEXA value indicates more pigmentation of the skin	Mexameter MX18
ERYTH (a.u.)	Erythma content, a larger ERYTH value indicates more Haem content of the skin	Mexameter MX18
b* (a.u.)	Skin yellowness, a larger b* value indicates more yellow skin tone	Colorimeter CL400
ITA (°)	Individual type angle, a composite indicator of skin colour from the CIE-L*a*b* colour system. A larger ITA value indicates lighter skin tone	Colorimeter CL400

Abbreviation	Factor meaning	Source
**Sect. 2 Abbreviations, meanings and sources of the meteorological factors**		
Air temperature (°C)	Air temperature, which is the average value of the data collected 28 days prior to testing	Ministry of Ecology and Environment: https://www.mee.gov.cn/
RH (%)	Relative humidity, which is the average value of the data collected 28 days prior to testing	
Wind speed (m/s)	Wind speed, which is the average value of the data collected 28 days prior to testing	
Sunshine duration (h)	Sunshine duration, which is the average value of the data collected 28 days prior to testing	
**Sect. 3 Abbreviations, meanings and sources of the air pollution data**		
AQI	Air quality index, which is the average value of the data collected 28 days prior to testing	Ministry of Ecology and Environment: https://www.mee.gov.cn/
PM_2.5_ (μg/m^3^)	Fine particulate matter, which is the average value of the data collected 28 days prior to testing	
PM_10_ (μg/m^3^)	Breathable particulate matter, which is the average value of the data collected 28 days prior to testing	
SO_2_ (μg/m^3^)	Sulfur dioxide, which is the average value of the data collected 28 days prior to testing	
CO (mg/m^3^)	Carbon monoxide, which is the average value of the data collected 28 days prior to testing	
NO_2_ (μg/m^3^)	Nitrogen dioxide, which is the average value of the data collected 28 days prior to testing	
O_3__8 h (μg/m^3^)	Ozone, which is the average value of the data collected 28 days prior to testing	

**Figure 1:**
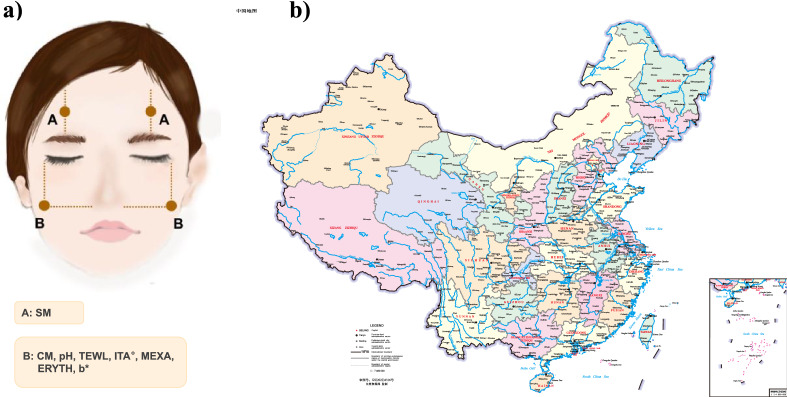
Overall skin physiological index test information. Test locations of skin physiological indexes (**a**). Seven cities located in mainland China: Beijing (BJ, North China, temperate monsoon climate) (39° N, 116° E), Shanghai (SH, East China, subtropical monsoon climate) (31° N, 121° E), Guangzhou (GZ, South China, subtropical monsoon climate) (23° N, 113° E), Chengdu (CD, Southwest China, subtropical monsoon climate) (30° N, 104° E), Shenyang (SY, Northeast China, temperate monsoon climate) (41° N, 123° E), Xi’an (XA, Northwest China, temperate monsoon climate) (34° N, 108° E) and Wuhan (WH, Central China, subtropical monsoon climate) (30° N, 114° E) ((**b**), drawing number: GS(2022)4316, the Ministry of Natural Resources of the People’s Republic of China).

China is a vast country with a wide range of latitudes and longitudes, which can be split into North China, Central China, East China, South China, Southwest, Northeast and Northwest according to the general geographical division in public health studies^5,6^. Due to differences in geographical locations, natural conditions and living habits, the skin characteristics of people are significantly different^7–9^. Dr. Liu measured skin colour characteristics in female volunteers from Guangzhou, Shanghai, Chengdu and Beijing, China between 2006 and 2007^10,11^. Kim et al. analysed the facial skin status, elasticity and wrinkles of female volunteers from Beijing, Shanghai, Guangzhou and Wuhan^12^. However, there are few studies for the overall regional facial skin status portrayal to our knowledge. The existing studies on skin status in different regions of China are not detailed and precise, with the insufficient scale and scope of non-invasive skin evaluation, and the research indicators mostly focusing on facial skin ageing and skin tone^10–12^. Thus, seven typical cities in each region were selected (Beijing, Wuhan, Shanghai, Guangzhou, Chengdu, Shenyang and Xi'an) based on the city’s geographical location, political, economic and cultural influence, in order to measure and describe the facial skin status of Chinese women in the different regions (Fig. 1b).

Differences in sex^13^, age^14^, geographical environments^15–18^, and lifestyles^19–21^, among other factors, will lead to different facial skin characteristics^22^. However, the association of facial skin status with environmental factors and individual lifestyles is unclear. Current studies have concerned on the molecular, cellular and microbiome levels^23–25^, with a few cross-sectional or cohort studies based on the Chinese population, and most of which focus on facial skin ageing and inflammatory responses^26–28^. In this study, the volunteers were limited to women aged 22–42 years to obtain a comprehensive understanding of the skin status of young and middle-aged women in China. The damage to the skin caused by sunshine exposure^29^, especially ultraviolet rays has become a consensus, which has been validated in southern Chinese women^30^. Liu et al. found the associations between skin status and climatic factors in the Shanghai population, such as temperature and relative humidity^31^. There is also consensus that air pollution can affect skin status, such as skin colour and tone^27^. Therefore, we selected four meteorological indicators including temperature, relative humidity (RH), sunshine duration and wind speed in conjunction with air quality index (AQI) data and observation data of six major pollutants including fine particulate matter (PM_2.5_), breathable particulate matter (PM_10_), sulfur dioxide (SO_2_), nitrogen dioxide (NO_2_), carbon monoxide (CO), and ozone (O_3__8h)^32^ to explore the association of facial skin status with environmental factors (obtained from the Ministry of Ecology and Environment: https://www.mee.gov.cn/). In addition, individual lifestyles^33^ also have an impact on skin status, such as cosmetic habits^34,35^, diet^36^, etc. Therefore, a questionnaire (Table S1) was administered to explore the association between the volunteers' facial skin status and individual lifestyles.

**Figure 2:**
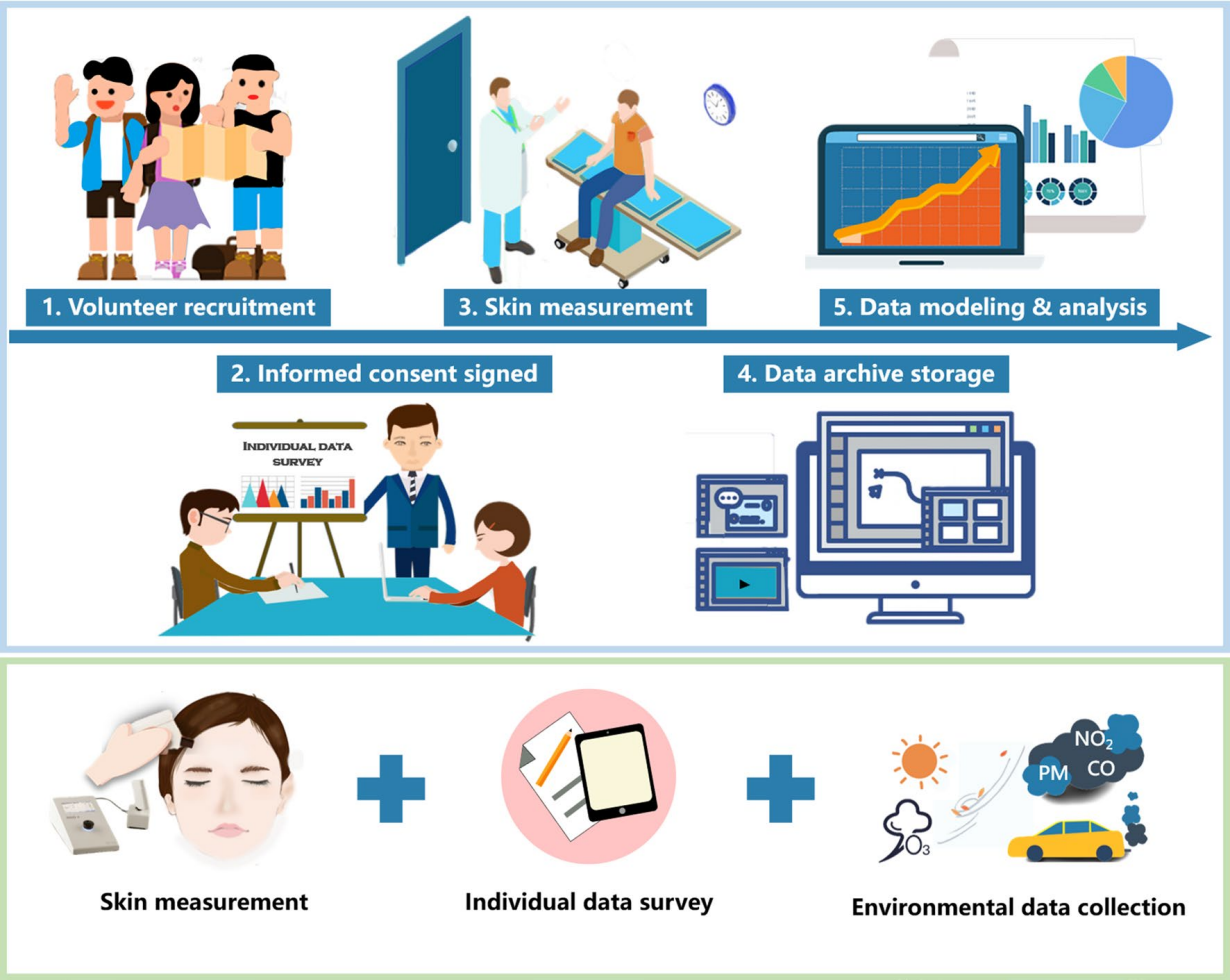
Research process of the multidimensional skin status study.

We designed this multidimensional skin status study based on methods, theories, examples, and developments related to noninvasive skin assessment (Fig. 2). In this study, we measured 8 facial skin parameters from 1092 Chinese females, collected environmental data from 7 cities in China and investigated the individual lifestyles of all the volunteers. The aim of this multidimensional study was to obtain baseline data on facial skin parameters, compare regional-related and environmental-related skin characteristics, and analyze the impact of different individual lifestyles on the facial skin barrier and tone to establish a facial skin status map of Chinese females to provide guidance for regional skincare.

## Materials and methods

We conducted a randomized recruitment trial in 7 cities between August 2019 and December 2019. The study was approved by the Ethics Committee of Xiyuan Hospital of the China Academy of the Chinese Medical Sciences (2019XL013-2) and was registered in the Chinese Clinical Trial Registry (registration number: ChiCTR1900025405). All methods were carried out in accordance with relevant guidelines and regulations. All healthy volunteers provided written consent for participation before enrolment. All documents clearly expressed the study objectives; therefore, all subjects were informed before signing the informed consent form.

### Subjects

A total of 1092 young Chinese women aged 22–42 years who lived in 7 cities in China for more than half a year were recruited by contract research institutions (CROs) through advertisements. To ensure an even overall age distribution of the volunteers in the seven-year circle, 52 volunteers in each age group were recruited in each city.

The exclusion criteria were as follows: (a) Menstruation, pregnant or breast-feeding women; (b) used hormone drugs or received anti-immunologic therapy within 1 month or during the study period; (c) undergone cosmetic surgery, cosmetic treatment, tattooing, maintenance, spotted nevus, facial fine-tuning, or cosmetic needle injection; (d) severe systemic, immunodeficiency or autoimmune diseases; (e) obvious skin allergy symptoms, facial damage, swelling or scars; (f) having a cold, headache or fever on the test day; (g) lack of consent, incomplete information or participating in other clinical trials.

### Protocol

Taking into account China’s administrative, climatic and geographical factors, the following 7 cities were selected to recruit subjects (Fig. 1b, Table 2): The test period ranged from 2019.08.21 to 2019.11.03, and individuals from the 7 cities were tested in descending latitude order (from Shenyang to Guangzhou) to eliminate the influence of season on temperature. All subjects attended a single visit after recruitment (approximately 1.5 h) at their corresponding CRO, at which time dermatologists and technicians performed measurements. Participants were asked to visit with bare facial skin, i.e., free from the application of any cosmetic product for 1 h, other than their routine cleaning procedure and/or products.

**Table 2:** Overall statistics of all participants.

Cities	All	Shenyang	Xi’an	Beijing	Chengdu	Wuhan	Shanghai	Guangzhou
**Sect. 1 Skin parameter collection period in each city**								
Period	2019.08.21–2019.11.03	2019.08.21–2019.08.26	2019.08.31–2019.09.05	2019.09.10–2019.09.18	2019.09.23–2019.09.28	2019.10.09–2019.10.14	2019.10.19–2019.10.24	2019.10.29–2019.11.03

Age stage	All	Shenyang	Xi’an	Beijing	Chengdu	Wuhan	Shanghai	Guangzhou
**Sect. 2 Statistics of all participants in all age groups by city**								
22–28	366	53	52	52	52	52	52	53
29–35	362	51	52	52	52	52	52	51
36–42	364	52	52	52	52	52	52	52
**Sect. 3 Descriptive analysis of age and skin physiological indexes in all volunteers by city (X ± SD)**								
Age	31.98 ± 5.86	32.13 ± 5.86	31.75 ± 5.87	31.91 ± 5.89	32.08 ± 6.23	31.88 ± 5.89	32.24 ± 5.96	31.88 ± 5.39
CM (a.u.)	60.01 ± 11.61	53.45 ± 9.17	58.09 ± 9.59	65.27 ± 8.79	59.98 ± 10.74	69.77 ± 10.11	55.97 ± 11.92	57.51 ± 11.95
TEWL (g/m^2^/h)	15.04 ± 4.29	15.11 ± 3.99	14.45 ± 2.86	15.34 ± 4.20	12.42 ± 3.45	14.93 ± 4.59	16.27 ± 4.55	16.74 ± 4.71
SM (μg/cm^2^)	73.11 ± 47.52	127.87 ± 66.23	76.33 ± 37.48	76.65 ± 40.73	64.64 ± 33.82	62.21 ± 33.81	50.06 ± 29.85	54 ± 33.62
pH	5.45 ± 0.50	5.39 ± 0.48	5.28 ± 0.45	5.42 ± 0.33	5.25 ± 0.47	5.57 ± 0.44	5.58 ± 0.55	5.63 ± 0.58
MEXA (a.u.)	166.64 ± 29.20	171.42 ± 26.89	169.65 ± 32.57	166.24 ± 27.96	167.02 ± 28.55	167.02 ± 24.62	150.89 ± 29.13	174.25 ± 28.72
ERYTH (a.u.)	296.36 ± 56.24	294.82 ± 50.86	320.18 ± 59.84	294.22 ± 59.54	312.1 ± 54.02	293.73 ± 53.21	295.33 ± 50.34	264.13 ± 48.85
ITA (°)	49.46 ± 8.25	51.53 ± 6.18	52.91 ± 6.08	52.70 ± 6.68	55.21 ± 5.78	47.11 ± 9.35	44.22 ± 6.78	42.54 ± 7.17
b* (a.u.)	12.68 ± 2.26	11.48 ± 1.62	11.88 ± 1.74	11.80 ± 1.67	12.98 ± 1.94	11.61 ± 1.78	14.19 ± 2.13	14.79 ± 2.25

### Noninvasive facial skin measurements

All measurements were taken with various probes attached to an MPA580 multiprobe adaptor system connected to a laptop (Table 1). SM test location was the intersection of the extension lines perpendicular to the midpoint of the eye and parallel to the midpoint of the forehead. Other skin status test (CM, pH, TEWL, ITA°, MEXA, ERYTH, b*) locations were the intersection points of the extension lines perpendicular to the angle of the eye and parallel to the lower part of the nasal wing. Symmetrical parts of the left and right sides of the face were tested, and final data obtained from 6 tests were averaged (Fig. 1a).

### Individual lifestyle survey

Volunteers were asked to fill out a Chinese paper questionnaire with a total of 62 questions divided into six parts: basic information, physical and mental health, past medical history, skin allergy history, cosmetic usage habits, and living habits (Table S1). Firstly, we excluded questionnaires missing values. Secondly, we eliminated questionnaires with obvious logical errors. (For example, multiple options were selected in single choice questions, or options with opposite meanings were selected simultaneously in multiple choice questions.) All excluded individual questionnaires were set to blank value in the analysis.

### Environmental data collection

In the normal epidermis, it takes 14 days for a keratinocyte cell to travel from the basal layer to the stratum corneum and 14 days to passage through the stratum corneum^37–40^ (Keratinization cycle). Considering that the metabolic cycle of keratinocytes is 28 days, the environmental data, including meteorological factors and pollutant data, of each city 28 days before testing were collected, and the impacts on skin status were analysed. Meteorological factors included air temperature, RH, sunshine duration, and wind speed; pollutant-related indicators included AQI, PM_2.5_, PM_10_, SO_2_, CO, NO_2_, and O_3__8 h contents (Table 1). All data were obtained from the Ministry of Ecology and Environment (https://www.mee.gov.cn/).

### Statistical analysis

#### ANOVA and Spearman rank correlation analysis

Significant differences in skin parameters among cities were calculated by one-way ANOVA and pairwise comparisons (IBM SPSS 25.0). The statistical tests were two-tailed, with a significance level of 0.05 (*P* < 0.05). Correlations between environmental data and skin physiological index data were calculated by Spearman rank correlation analysis^41^.

#### Principal component analysis (PCA) and the optimal subset regression model

The questionnaire information was analysed with mathematical modelling. First, the question options were scored (with 0 as the standard state; higher scores indicated worse health status); regular expression was used to convert the multiple-choice questions into 0/1 format data. Then, the principal components of the 62-item questions were extracted by PCA. The optimal subset regression model was used to analyse the principal components affecting each skin physiological parameter (R 4.0.4). Finally, the effects of the five questionnaire modules on each physiological skin parameter were analysed. Specific modeling process in Supplementary Materials 2.1–2.6.

### Ethics statements

The study was approved by the Ethics Committee of Xiyuan Hospital of the China Academy of the Chinese Medical Sciences (2019XL013-2) and was registered in the Chinese Clinical Trial Registry (registration number: ChiCTR1900025405). All methods were carried out in accordance with relevant guidelines and regulations.

## Results

### Skin status and environmental factor analysis

#### Skin barrier and skin tone data analysis of 7 cities in China

**Figure 3:**
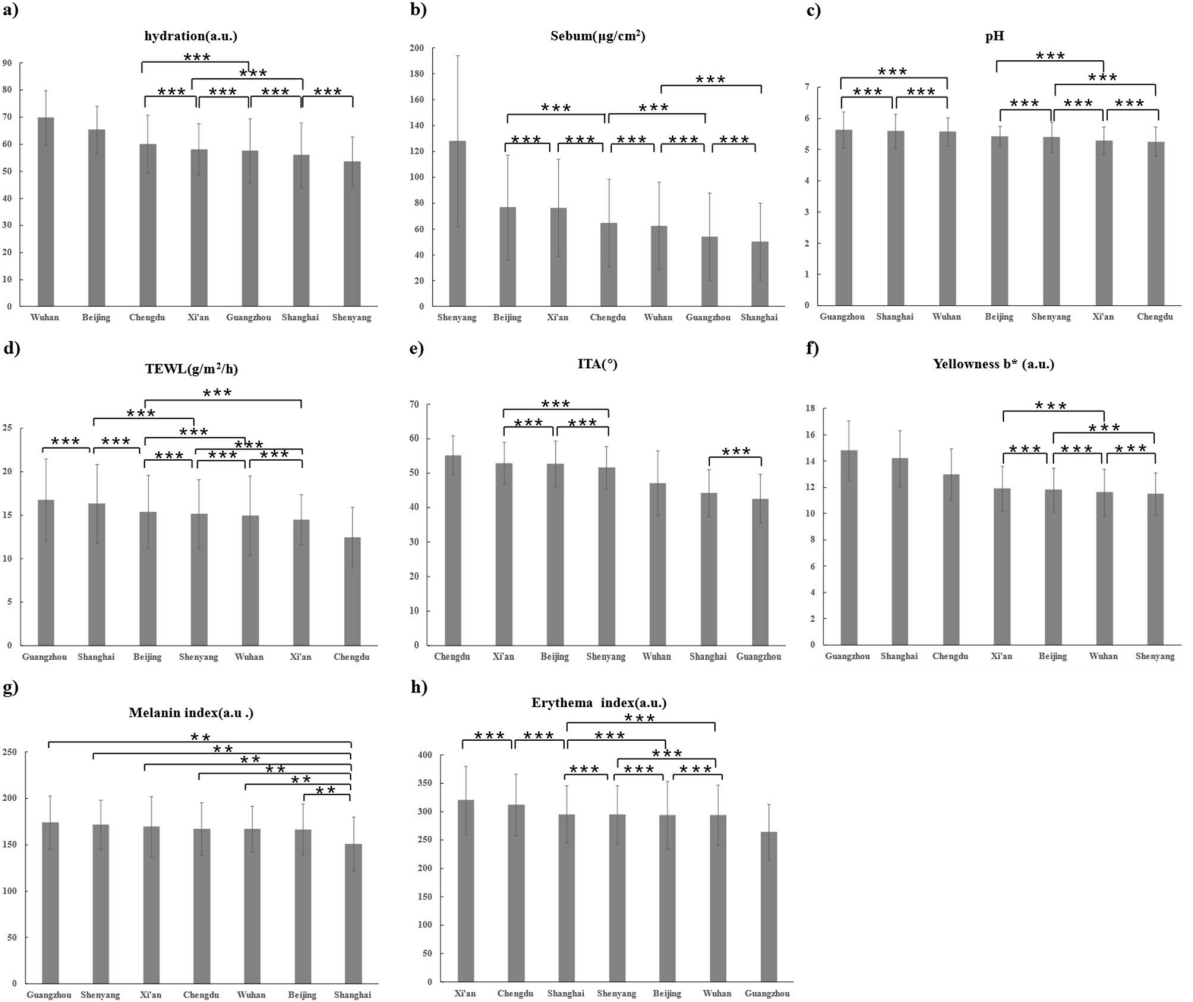
Comparison of skin physiological index differences among participants from seven cities. Skin hydration content (**a**); skin sebum content (**b**); skin pH value (**c**); skin TEWL value (**d**); individual typological angle ITA (**e**); skin yellowness (b*) value (**f**); skin melanin index value (**g**); skin erythema index value (**h**). **Indicates a significant difference between the two cities (*P* < 0.05). ***Indicates no significant difference between the two cities (*P* > 0.05).

The CM value of participants in the Wuhan was highest, with a significant difference, followed by the Beijing; the CM value of participants in the Shenyang was lowest (Fig. 3a, Table 2). The SM value of participants in the Shenyang was significantly higher than those in Shanghai (Fig. 3b, Table 2). The skin pH of volunteers in Guangzhou was the highest. The skin pH of volunteers in Chengdu was the lowest (Fig. 3c, Table 2). The TEWL value of participants in Chengdu was significantly lower, and the skin barrier function was significantly better than those of participants in the other cities. The TEWL value of participants in Guangzhou was significantly higher, and the skin barrier function was significantly poorer (Fig. 3d, Table 2). Supplementary Materials 1.1 for specific data.

The skin ITA° value of participants in Chengdu was the largest, and their skin tone was significantly lighter; the skin ITA° values of participants in Shanghai and Guangzhou were small, and their skin tone was significantly darker (Fig. 3e, Table 2). The b* value of participants in Guangzhou was highest, followed by those in Shanghai and Chengdu. (Fig. 3f, Table 2). The MEXA value of participants in Shanghai was significantly lower than those in the other six cities, and the differences between those in the other cities were not significant (Fig. 3g, Table 2). The ERYTH value was significantly higher in Xi’an participants, followed by Chengdu participants; the ERYTH value was significantly lower in Guangzhou participants (Fig. 3h, Table 2). Supplementary Materials 1.1 for specific data.

#### Meteorological factors and air pollution data analysis of 7 cities in China

**Figure 4:**
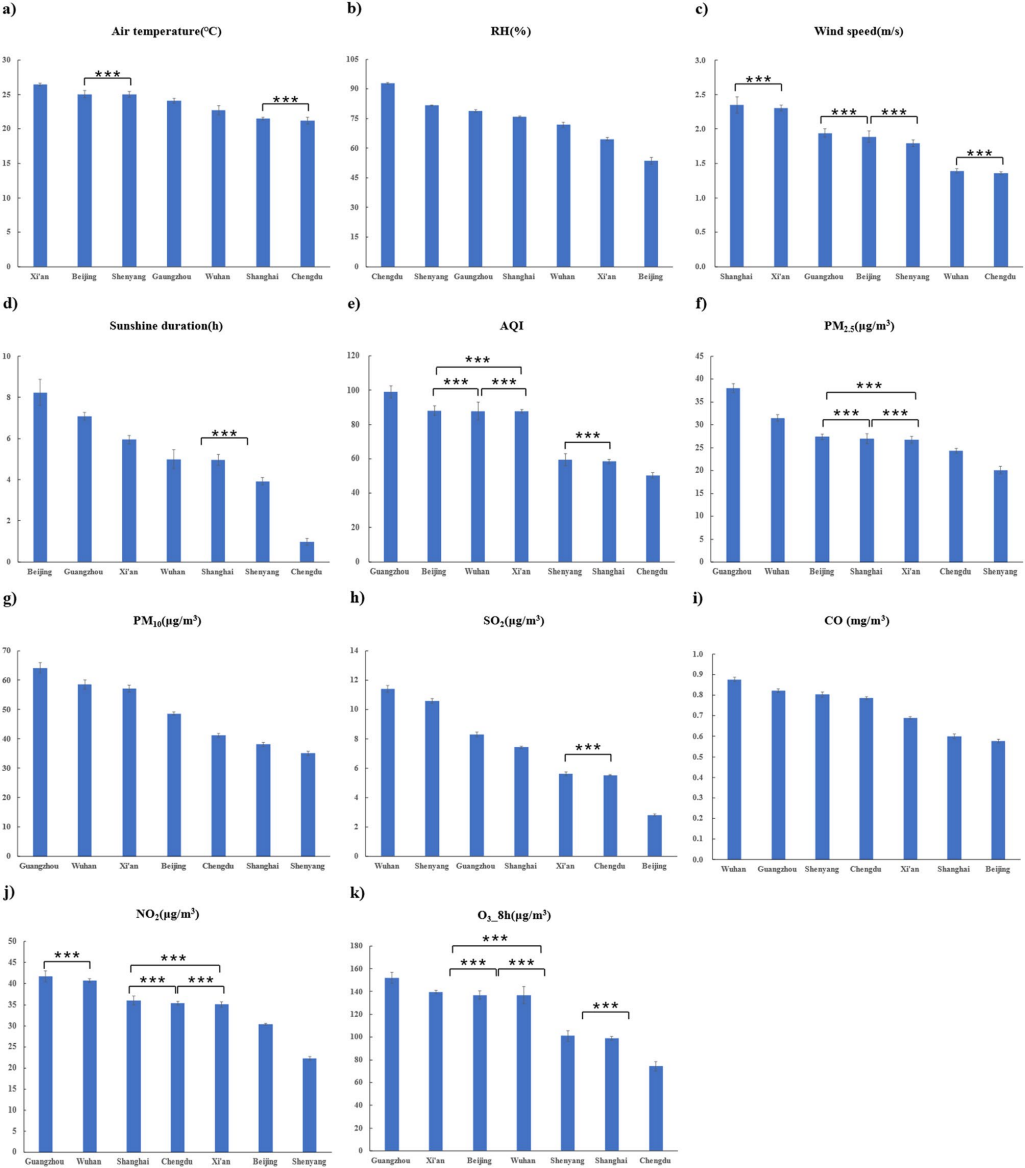
Comparison of environmental factors and air pollution indicators in seven cities. Air temperature (**a**); relative air humidity (RH) (**b**); wind speed (**c**). Sunshine duration (**d**); AQI (**e**); PM_2.5_ values (**f**); PM_10_ values (**g**); SO_2_ values (**h**); CO values (**i**); NO_2_ values (**j**); O_3__8 h values (**k**). **Indicates a significant difference between the two cities (*P* < 0.05). ***Indicates no significant difference between the two cities (*P* > 0.05). Figure without ** or ***indicates a significant difference between two cities.

The Xi’an (26.46) temperature was significantly higher, and the Shanghai (21.50) and Chengdu (21.22) temperatures were significantly lower (Fig. 4a, Table 3) than those in other cities. The results of RH were as follows: the pairwise differences between cities were all significant. The RH in Chengdu (92.90) was significantly higher, and that in Beijing (53.49) was significantly lower than those in the other cities (Fig. 4b, Table 3). The results for wind speed were as follows: there were significant differences between cities. Shanghai (2.35) had the fastest wind speed, followed by Xi’an (2.30); Wuhan (1.39) and Chengdu (1.36) had slower wind speeds than those in the other cities (Fig. 4c, Table 3). The results for sunshine duration were as follows: there was no significant difference between Wuhan and Shanghai, but there were significant differences between the other cities. Beijing (8.24) had the longest sunshine duration, followed by Guangzhou (7.07); Chengdu (0.97) has the shortest sunshine duration (Fig. 4d, Table 3).

**Table 3:** Descriptive analysis of normal environmental factors and air pollutants data in various cities (X ± SD).

Cities	Shenyang	Xi’an	Beijing	Chengdu	Wuhan	Shanghai	Guangzhou
Air temperature (°C)	25.01 ± 0.39	26.46 ± 0.19	25.03 ± 0.54	21.22 ± 0.48	22.73 ± 0.62	21.50 ± 0.19	24.10 ± 0.34
Relative air humidity (RH) (%)	81.59 ± 0.18	64.54 ± 0.84	53.49 ± 1.76	92.90 ± 0.42	71.77 ± 1.31	75.84 ± 0.49	78.76 ± 0.71
Wind speed (m/s)	1.80 ± 0.05	2.30 ± 0.04	1.89 ± 0.08	1.36 ± 0.02	1.39 ± 0.03	2.35 ± 0.12	1.94 ± 0.06
Sunshine duration (h)	3.92 ± 0.18	5.95 ± 0.20	8.24 ± 0.64	0.97 ± 0.16	4.99 ± 0.46	4.96 ± 0.26	7.07 ± 0.20
AQI	59.58 ± 3.49	87.78 ± 1.08	88.10 ± 2.83	50.39 ± 1.54	87.81 ± 5.30	58.51 ± 1.18	99.08 ± 3.53
PM_2.5_ (μg/m^3^)	20.12 ± 0.76	26.72 ± 0.77	27.39 ± 0.59	24.33 ± 0.47	31.50 ± 0.64	26.96 ± 1.09	37.99 ± 0.95
PM_10_ (μg/m^3^)	35.10 ± 0.72	57.16 ± 1.12	48.59 ± 0.55	41.27 ± 0.60	58.50 ± 1.58	38.13 ± 0.74	64.19 ± 1.72
SO_2_ (μg/m^3^)	10.58 ± 0.17	5.61 ± 0.13	2.80 ± 0.07	5.51 ± 0.04	11.40 ± 0.25	7.44 ± 0.07	8.30 ± 0.15
CO (mg/m^3^)	0.80 ± 0.01	0.69 ± 0.01	0.58 ± 0.01	0.79 ± 0.01	0.88 ± 0.01	0.60 ± 0.01	0.82 ± 0.01
NO_2_ (μg/m^3^)	22.24 ± 0.42	35.12 ± 0.63	30.41 ± 0.18	35.38 ± 0.40	40.76 ± 0.43	36.02 ± 1.09	41.72 ± 1.28
O_3__8 h (μg/m^3^)	101.10 ± 4.61	139.73 ± 1.26	136.93 ± 3.72	74.33 ± 4.15	136.92 ± 7.68	98.85 ± 1.48	151.99 ± 4.62

There were significant differences between Guangzhou and Wuhan (*P* = 0.05). Guangzhou had the highest AQI value (99.08), and Chengdu had the lowest AQI value (50.39) (Fig. 4e, Table 3). The results for PM_2.5_ were as follows: there was no significant difference between Xi’an and Beijing, Xi’an and Shanghai, and Beijing and Shanghai, while the differences between the other cities were significant. The Guangzhou PM_2.5_ (37.99) concentration was the highest, and the Shenyang PM_2.5_ (20.12) concentration was the lowest (Fig. 4f, Table 3). The results of PM_10_ were as follows: all the cities had significant pairwise differences. The Guangzhou PM_10_ (64.19) concentration was the highest, and the Shenyang PM_10_ (35.10) concentration was the lowest (Fig. 4g, Table 3). The results of SO_2_ were as follows: the difference between Xi’an and Chengdu was not significant, while the differences between the other cities were significant. The Wuhan SO_2_ (11.40) concentration was the highest, and the Beijing SO_2_ (2.80) concentration was the lowest (Fig. 4h, Table 3). The results of CO were as follows: all pairwise differences were significant for the seven cities. The Wuhan CO (0.88) concentration was the highest, and the Beijing CO (0.58) concentration was the lowest (Fig. 4i, Table 3). The results of NO_2_ were as follows: there were significant differences between the cities. The Guangzhou NO_2_ (41.72) concentration was the highest, followed by Wuhan (40.76); the Shenyang NO_2_ (22.24) concentration is the lowest (Fig. 4j, Table 3). The results for O_3__8 h were as follows: there was no significant difference between Xi’an and Beijing, Xi’an and Wuhan, Beijing and Wuhan, and Shenyang and Shanghai, while the difference between the other two was significant. The Guangzhou O_3__8 h (151.99) concentration was the highest, and the Chengdu O_3__8 h (74.33) concentration was the lowest (Fig. 4k, Table 3).

### Correlation analysis of environmental data and skin data

#### Correlation analysis of meteorological factors and skin data

Spearman correlation analysis showed that the 4 skin barrier indicators were correlated with meteorological factors, as follows: the CM value was negatively correlated with RH and wind speed; the TEWL value was positively correlated with sunshine duration and wind speed; the SM value was positively correlated with temperature; and skin pH was negatively correlated with temperature. The 4 indicators of skin tone level were correlated with meteorological factors as follows: the skin ITA° value was negatively correlated with wind speed, and skin b* was negatively correlated with temperature; the MEXA value was positively correlated with temperature (Table 4).

**Table 4:** Correlation analysis between environmental data and skin physiological index data (X ± SD).

Skin physiological indexes	Hydration	Sebum	pH	TEWL	ITA	Yellowness b*	Melanin index	Erythema index
Air temperature (°C)	− 0.144	0.534**	− 0.319*	0.109	0.058	− 0.459**	0.409**	0.162
RH (%)	− 0.446**	− 0.040	− 0.014	− 0.296	0.092	0.260	0.029	− 0.092
Wind speed (m/s)	0.243	− 0.096	0.083	0.513**	− 0.253	0.019	0.139	− 0.235
Sunshine duration (h)	− 0.390*	− 0.054	− 0.017	0.494**	− 0.397*	0.287	− 0.039	− 0.004
AQI	0.248	− 0.136	0.126	0.522**	− 0.364*	− 0.042	0.334*	− 0.276
PM2.5 (μg/m^3^)	0.446**	− 0.551**	0.341*	0.453**	− 0.529**	0.309	0.073	− 0.427**
PM10 (μg/m^3^)	0.498**	− 0.364*	0.155	0.220	− 0.264	0.140	0.296	− 0.140
SO2 (μg/m^3^)	− 0.115	− 0.087	0.270	0.232	− 0.440**	− 0.103	0.170	− 0.290
CO (mg/m^3^)	0.075	− 0.090	0.155	− 0.028	− 0.224	− 0.088	0.321*	− 0.218
NO2 (μg/m^3^)	0.327*	− 0.684**	0.343*	0.207	− 0.492**	0.520**	0.041	− 0.272
O3_8 h (μg/m^3^)	0.209	− 0.109	0.122	0.490**	− 0.332*	− 0.045	0.332*	− 0.268

*Indicates a significant correlation at the 0.05 level.

**Indicates a significant correlation at the 0.01 level.

In summary, an increase in wind speed or sunshine duration is accompanied by an increase in TEWL value, i.e. poorer skin barrier function; a decrease in wind speed is accompanied by an increase in CM and ITA values, i.e. whiter and more hydrated skin. The increase in temperature is accompanied by an increase in SM, MEXA value and a decrease in pH value, i.e. oilier, darker and more acidic skin. Notably, we also observed that an increase in RH was accompanied by a decrease in CM value.

#### Correlation analysis of air pollutant elements and skin data

The 4 skin barrier indicators were correlated with air pollutants. The CM value was positively correlated with PM_2.5_, PM_10_, and NO_2_ contents, whereas the SM value was negatively correlated with PM_2.5_, PM_10_, and NO_2_ contents. In addition, skin pH was negatively correlated with PM_2.5_ and NO_2_ contents. The TEWL value was positively correlated with the AQI, PM_2.5_ content, and O_3__8 h content. The 4 skin tone indicators were correlated with air pollutants. The ITA value was negatively correlated with the AQI, PM_2.5_ content, SO_2_ content, NO_2_ content, and O_3__8 h content. Regarding skin b*, a positive correlation with NO_2_ content was observed. The MEXA value was positively correlated with the AQI, CO content, and O_3__8 h content, while the skin ERYTH value was negatively correlated with PM_2.5_ content (Table 4).

In summary, increased AQI value was accompanied by decreased ITA values, increased TEWL and MEXA value, i.e. poor barrier function and darker skin. Increased PM_2.5_/PM_10_ or NO_2_ contents were accompanied by increased CM and TEWL values, as well as decreased SM and pH values, i.e. less Sebum secretion, more acidic but more hydrated skin.

### The impact of individual lifestyle on the skin barrier and skin tone based on PCA

We analysed the effects of individual lifestyle on skin barrier and skin tone based on PCA. In order to differentiate the influence of the questionnaire questions on each skin indicator, the 62 questions were categorised according to the content of the questions into five categories (physical and mental health, skin allergy history, cosmetic use, lifestyle habits, and past medical history). Finally, a correlation model between the various skin physiological indicators and the five categories of questions was developed and the effect of the five questionnaire modules on the eight skin parameters was analysed (Supplementary Materials 2.1–2.6).

**Figure 5:**
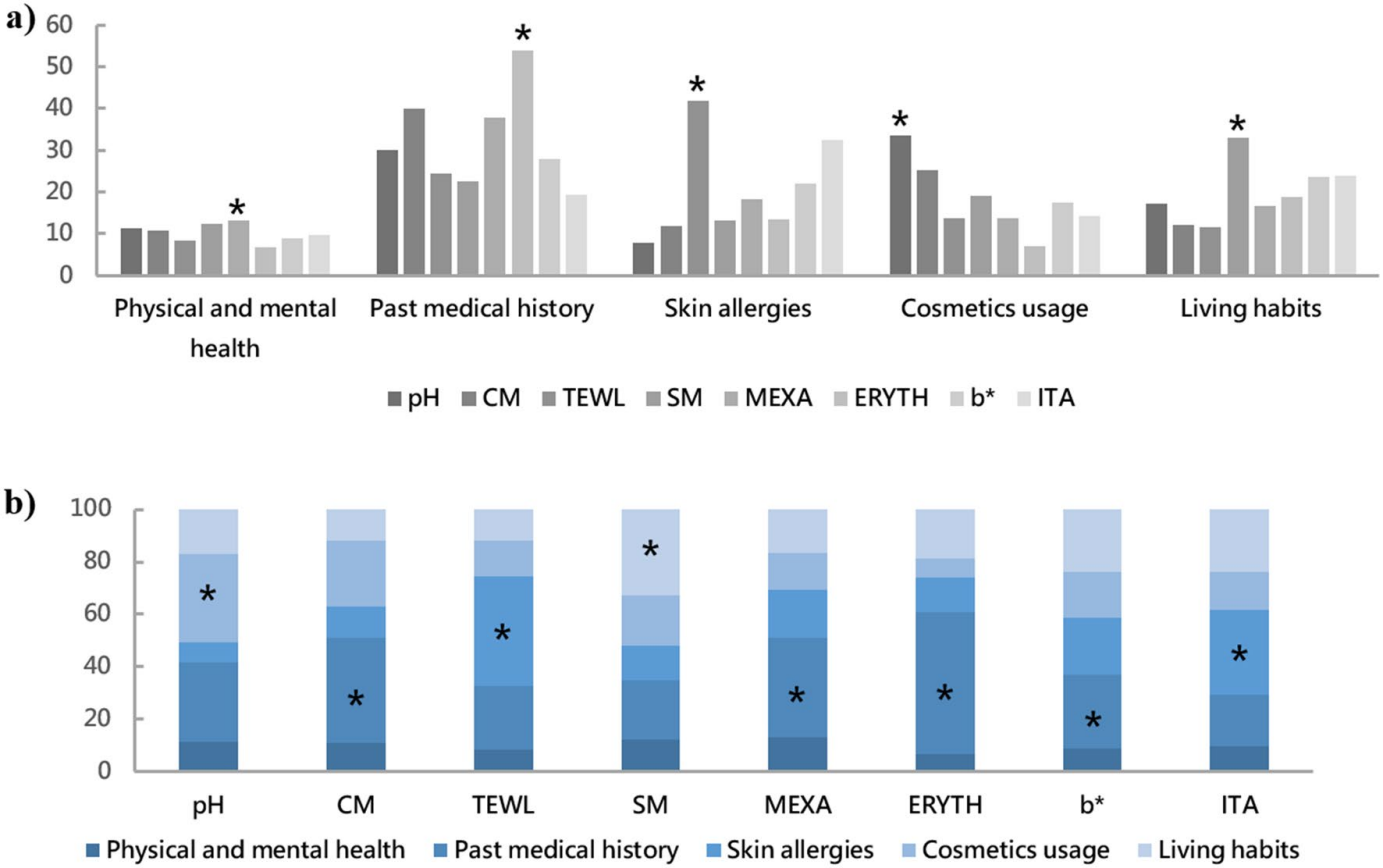
Lateral impacts of individual lifestyle on each skin parameter (%) (**a**). Longitudinal impacts of individual lifestyle on each skin parameter (%) (**b**). *Indicates the maximum value of this group (the Supplementary Materials 1.1 for specific data).

Overall, cosmetic usage had the greatest impact on skin pH and CM values, followed by past medical history (Fig. 5). Skin allergies had the greatest impact on the transcutaneous water loss. Living habits had the greatest impact on SM value. Physical and mental state had a minimal impact on the skin barrier, as well as skin tone (Fig. 5). Skin allergy had the largest impact on the ITA value, and past medical history had the largest impacts on the other indicators of skin tone, such as MEXA, ERYTH and b* values.

## Discussion

### Facial skin barrier and skin tone render regional differences in China

We divided skin indicators into “skin barrier function” and “skin tone”. With regard to skin barrier function, the regions showed a western-others-southern trend. Women living in two western cities (Chengdu, Xi’an) had a better skin barrier, as indicated by high hydration and sebum secretion, together with a weakly acidic pH value. Those women who live in South China (Guangzhou) had a poor skin barrier, as indicated by highest transcutaneous water loss, lowest sebum secretion and a neutral pH value. Other regions did not reveal significant barrier characteristics.

Compared to the western regions (Chengdu, Xi’an), Guangzhou has the worst circumstance of air pollution and longer sunshine duration (longest in Beijing). From the perspective of pollution in Guangzhou, the particulate matter and nitrogen dioxide had the largest effects^42^. Our results complement the association between pollution conditions and the skin barrier in Guangzhou. Kim et al. investigated the effects of PM_2.5_ in South Korea on filaggrin and skin barrier function in vitro and in vivo^43^. They found that PM_2.5_ exposure compromised skin barrier function, resulting in increased transcutaneous water loss, similar to the results in Guangzhou. PM_2.5_-induced TNF-α caused filaggrin deficiency in the skin and subsequently induced skin barrier dysfunction, which may be one of the reasons for the poor skin function of women in Guangzhou. There is no doubt that natural and sun-induced ageing of human skin is inevitable^44^. In recent years, studies on the effects of chronic UVB irradiation on the integrity of the skin barrier (transcriptomic^45^, lipidomic^46^, animal models^47^) have been validated. As most of the UVB light is absorbed in the epidermis^48^, the outermost cell layers, the stratum corneum, and the stratum granulosum were most affected, resulting in a breakdown of the epidermal physical barrier (protects against radiation and tries to maintain of permeability)^49^. Santiago et al. found that skin received chronic UVB radiation had a higher skin surface pH value, with the permeability barrier and stratum corneum hydration severely compromised in a Murine Model of Cutaneous Field Cancerization^50^. This is consistent with our study that female skin exhibits high transcutaneous water dissipation, low hydration and neutral pH in Guangzhou with longer sunshine duration. Zheng et al. found that cathepsin D decreased in chronic photodamaged skin. Nevertheless, cathepsin D plays an important part in maintaining a normal skin barrier, which may be one of the reasons for the poor skin function of women in Guangzhou^51^.

The rank distribution of skin tone level was consistent with skin barrier (western-others-southern). In Guangzhou, the darker skin tone was easy to observe. Lighter skin tones were observed in relatively well-protected regions (Chengdu, Xi’an). The color of the skin is determined by the presence of several major chromophores, melanin and in some cases, oxy-/deoxyhemoglobin and carotenoids^52^. UVA induces immediate pigment darkening, persistent pigment darkening, and delayed tanning; UVB is an effective spectrum to induce erythema, which is followed by delayed tanning; visible light has been shown to induce erythema and a tanning response in dark skin^53,54^. From the perspective of pigment distribution, women in the western regions (Chengdu, Xi’an) received lower sunshine duration and ultraviolet rays with lower melanin content, while the melanin content in Guangzhou was the highest due to longer sunshine duration. But in this study, we did not observe significant differences in erythema index between the Guangzhou region and the western region. Kollias et al. found that ultraviolet radiation could convert oxyhemoglobin and deoxyhemoglobin stoichiometrically into methemoglobin and a met-like product, respectively, but not on all volunteers tested^55^. In a Case of Methemoglobinemia, the patient was found with blue skin discoloration instead of usually pink^56^. It is uncertain whether this conversion is associated with the low erythema index of the Guangzhou volunteers.

### Meteorological factors affect skin status, and pollution triggers skin issues

In this study, we illustrated that meteorological factors can affect skin status. Under normal humidity conditions, temperature affects various functions of the skin barrier^57^. Elevated temperature was significantly associated with increased sebum secretion, decreased pH, and increased melanin content. Wearing mask makes the positive effect of temperature on sebum production more apparent^58,59^. Engebretsen et al. reviewed the negative effect of low humidity, low temperatures and different seasons on the skin barrier, and concluded that low humidity and low temperatures lead to a general decrease in skin barrier function and increased susceptible towards mechanical stress^60^. Notably, we found that an increase in environmental relative humidity was accompanied by an increase in transcutaneous water loss. Vyumvuhore et al. found that lipid organization as well as protein deployment was optimal at intermediate RH values (around 60%) ex vivo Raman spectroscopy analysis, which correspond to the maximum of Stratum corneum water binding capacity. Moreover, the contributions of totally bound water were not vary with humidity, and increased content of unbound water in the SC induces disorder in the structures of lipids and proteins^61^. This may explain to some extent the positive correlation between relative humidity and transcutaneous water loss.

Air pollution triggers skin issues, such as skin pigmentation and the deconstruction of skin barrier. The AQI characterizes only the degree of pollution and not the concentration of specific pollutants^59^. Each pollutant was converted to an AQI value according to different target concentration limits during the evaluation^62^. Thus, we disassembled the various components of the AQI and analysed their correlations with skin indicators. The most obvious factors affecting the skin were the concentrations of PM_2.5_ and NO_2_. Compared with SO_2_, NO_2_ showed a stronger correlation with the skin indicators^63^. The increase in NO_2_ content was accompanied by a decrease in skin oil content and an increase in moisture, which causes reduced acidity of the skin^64,65^. However, the skin tone darkens with the ITA° and becomes more yellow. PM_2.5_ induces apoptosis, oxidative stress injury and melanin metabolic disorder in human melanocytes^66^. The results showed that an increase in PM_2.5_ content was accompanied by an increase in moisture content and loss; however, the oil content was significantly reduced. This result was also accompanied by a decrease in skin acidity as well as decreased value of erythema index and a darker skin tone^67^. Shi et al. illustrated that PM2.5 can induce melanogenesis in vivo/vitro by regulating TYR, TYRP1, TYRP2, and MITF expression via AhR/MAPK signaling activation. Furthermore, PM2.5 increased α-MSH paracrine levels, which in turn promote hyperpigmentation^68^. Moreover, UVB induced the activation of AhR during long sunshine duration, leading to a series of melanin increases and skin barrier disruptions^69^. These studies at the molecular level may explain the skin status differences between the Guangzhou and western regions.

### Individual lifestyle affects skin barrier and skin tone

In this study, we found that cosmetic usage had the greatest impact on skin pH and hydration content (CM), followed by past medical history. The positive impact of beauty habits on the skin has been proven in Korea and in the Chinese female population in China. Lee et al. found that using sunscreen every day, wearing base makeup daily, and using moisturizers improved hydration, transcutaneous water loss, and elasticity significantly after adjusting for age and region^34,35^. Transcutaneous water loss (TEWL) was most affected by skin allergies, which is the most widely used objective measurement for assessing the barrier function of skin in healthy individuals and also patients with skin diseases that are associated with skin barrier dysfunction, such as atopic dermatitis^70^. A significant increase in TEWL is observed in children with skin allergic diseases such as atopic dermatitis^71^. Allergic disorders such as atopic dermatitis are strongly associated with an impairment of the epithelial barrier^72^, in which tight junctions and/or filaggrin expression can be defective^73^. Living habits had the greatest impact on sebum secretion (SM). Bissonnette et al. found that variations in sleep patterns was associated with changes in sebum excretion in women^74^. Many studies have found the effect of diet on the amount of sebum secretion^36,75,76^. Nives Pondeljak provide a detailed review of stress-induced interactions of skin immune cells, hormones, and neurotransmitters^77^. Review by Passeron et al. mentioned that stress and sleep deprivation can lead to a pro-inflammatory state, which in turn affects the integrity of extracellular matrix proteins, especially collagen^78^. However, we found that the overall effect of physical and mental state on both skin barrier and color was minimal compared to other factors investigated. Skin allergies had the largest impact on the individual type angle (ITA), and past medical history had the largest impacts on the other indicators of skin tone (MEXA, ERYTH, b*). However, we estimate that this distribution of skin tone is more likely to be the result of environmental factors rather than individual lifestyles.

## Conclusions

There were regional differences in facial skin barrier characteristics and skin tone. An obvious difference was a better skin barrier in the western region, as indicated by high skin hydration and sebum secretion and a weakly acidic pH. According to the ITA value, a composite indicator of skin colour from the CIE-L*a*b* colour system, as the main grading standard^79^, lighter and darker skin tones were found in the western and southern regions, respectively. Under normal humidity conditions, temperature affects various functions of the skin barrier. When pollutant concentrations increase, they not only affect the barrier function of the skin but also cause obvious concomitant changes in skin tone. According to the analysis of individual lifestyle surveys, the overall effect of the use of cosmetics had the greatest impact on the pH and hydration of the skin. Skin allergies had the greatest impact on transcutaneous water loss and skin tone. Living habits had the greatest impact on sebum secretion.

In this study, we measured 8 facial skin parameters from 1092 Chinese females, collected environmental data from 7 cities in China and investigated the individual lifestyles of all the volunteers. The baseline data on facial skin parameters was obtained, regional-related and environmental-related skin characteristics was compared, and the impact of different individual lifestyles on the facial skin barrier and tone was analysed to establish a facial skin status map of Chinese females to provide guidance for regional skincare. All in all, this cross-sectional study of Chinese women in seven cities provides evidence of associations of facial skin status with environmental factors and individual lifestyles in a large population of Chinese people, and reviews a number of relevant studies with the molecular mechanisms. Regrettably, separate specific questions about individual lifestyles were not discussed in this study, and we only considered the association of five broad questions with skin status.

## Supplementary Information

Supplementary Information.

## Data Availability

The datasets used and/or analysed during the current study available from the corresponding author on reasonable request.
