# Supplementary material for: A cross-sectional study of Chinese women facial skin status with environmental factors and individual lifestyles
Source: Sci Rep. 2022 Oct 27;12:18110. doi: 10.1038/s41598-022-23001-6 (PMC9613773; doi:10.1038/s41598-022-23001-6)
Supplement: Supplementary file 1 — Supplementary Information. [file 41598_2022_23001_MOESM1_ESM.docx]

# Supplementary Material

## 1 Skin Status and environmental factor analysis

### 1.1 Skin barrier and skin tone data analysis of 7 cities in China

The results of skin hydration content were as follows: Wuhan (69.77)> Beijing (65.27)> Chengdu (59.98)> Xi’an (58.09)> Guangzhou (57.51)> Shanghai (55.97)> Shenyang (53.45). There were significant differences between all pairwise cities except for Chengdu and Guangzhou, Chengdu and Xi’an, Guangzhou and Shanghai, Guangzhou and Xi’an, Shanghai and Shenyang, and Shanghai and Xi’an (Figure 3a, Table 1). The results of skin sebum production were as follows: Shenyang (127.87)> Beijing (76.65)> Xi’an (76.33) Chengdu (64.64)> Wuhan (62.21)> Guangzhou (54)> Shanghai (50.06). There were significant differences between the other pairwise cities (Figure 3b, Table 1). The results of skin pH values were as follows: Guangzhou (5.63)> Shanghai (5.58)> Wuhan (5.57)> Beijing (5.42)> Shenyang (5.39)> Xi’an (5.28)> Chengdu (5.25). There were significant differences between pairwise cities (Figure 3c, Table 1). The results of skin TEWL values were as follows: Guangzhou (16.74)> Shanghai (16.27)> Beijing (15.34)> Shenyang (15.11)> Wuhan (14.93)> Xi’an (14.45)> Chengdu (12.42). There were significant differences between pairwise cities. The TEWL value of participants in Chengdu was significantly lower, and the skin barrier function was significantly better than those of participants in the other cities (Figure3d, Table 1).

The ITA° characterizes skin tone. The results for skin ITA° were as follows: Chengdu (55.21)> Xi’an (52.91)> Beijing (52.70)> Shenyang (51.53)> Wuhan (47.11)> Shanghai (44.22)> Guangzhou (42.54). There were significant differences between cities (Figure 3e, Table 1). The results of skin yellowness (b*) values were as follows: Guangzhou (14.79)> Shanghai (14.19)> Chengdu (12.98)> Xi’an (11.88)> Beijing (11.8)> Wuhan (11.61)> Shenyang (11.48). There were significant differences between cities(Figure 3f, Table 1). The results for skin melanin index values were as follows: Guangzhou (174.25)> Shenyang (171.42)> Xi’an (169.65)> Wuhan (167.02)= Chengdu (167.02)> Beijing (166.24)> Shanghai (150.89) (Figure 3g, Table 1). The results of skin erythema index values were as follows: Xi’an (320.18)> Chengdu (312.1)> Shanghai (295.33)> Shenyang (294.82)> Beijing (294.22)> Wuhan (293.73)> Guangzhou (264.13). There were significant differences between cities except for Beijing and Shanghai, Beijing and Shenyang, Beijing and Wuhan, Chengdu and Shanghai, Chengdu and Xi’an, Shanghai and Shenyang, Shanghai and Wuhan, Shenyang and Wuhan (Figure 3h, Table 1).

## 2 Skin parameter-individual lifestyle correlation analysis

### 2.1 Questionnaire preprocessing

The questionnaire information (Table S1) was analysed through mathematical modelling. First, the question options were scored (with 0 as the standard state; a higher score indicated, worse health); the regular expression was used to convert the multiple-choice questions into 0/1 format data (selected was 1, unselected was 0, and all contradictory options were set to 0). Then, the principal components of the 62 questions were extracted by PCA; the optimal subset regression model was used to analyse the principal components affecting each skin parameter (R 4.0.4). Finally, the effects of the five questionnaire modules on each skin parameter were analysed.

### 2.2 Correlation analysis of 62 questions

Cluster analysis of 62 survey questions revealed that the questions roughly clustered into 4 categories (Figure S1). However, cluster analysis cannot reveal the specific impacts of each type of problem response. Therefore, factor analysis was finally selected for follow-up analysis.

### 2.3 Factor analysis

According to the gravel chart, the broken line became flat after 14 factors. The selection of 14 main factors covered the information to be obtained by the questionnaire questions (Figure S2).

### 2.4 Optimal subset regression model

There are two common feature selection methods for linear regression models, namely, an optimal subset and stepwise regression. When using the optimal subset and stepwise regression for feature selection, the result obtained by the optimal subset was better than that obtained by stepwise regression, so the optimal subset was selected for subsequent analysis. Through the optimal subset analysis between each skin index and the principal components of the questionnaire, the principal components that had greater impacts on each skin index were determined (Figure S3, Table S2).

### 2.5 Correlation between skin parameters and questionnaire questions

To distinguish the influence of the questionnaire questions on each skin index, classification processing was carried out according to the questions. The 62 questions were divided into five categories to explore the effects of physical and mental health, skin allergies, cosmetic use, living habits, and past medical history on skin barrier and colour and to establish a correlation model between various skin parameters and the five categories of questions.

## 2.6 The impact of individual lifestyle on the skin barrier and skin tone based on PCA

The results of skin pH were as follows: cosmetic usage (33.50%) > past medical history (30.12%) > living habits (17.18%) > physical and mental health (11.41%) > skin allergy (7.79%). The results of skin moisture content were as follows: past medical history (40.07%) > cosmetic usage (25.12%) > living habits (12.07%) > skin allergies (11.83%) > physical and mental health (10.91%). The results of skin TEWL values were as follows: skin allergy (41.77%) > past medical history (24.38%) > cosmetic usage (13.62%) > living habits (11.72%) > physical and mental health (8.51%). The results of skin sebum production were living habits (32.92%) > past medical history (22.54%) > cosmetic usage (19.04%) > skin allergies (13.21%) > physical and mental status (12.29%) (Figure 5). Overall, cosmetic usage had the greatest impact on skin pH and hydration content, followed by past medical history. Skin allergies had the greatest impact on TEWL. Living habits had the greatest impact on sebum secretion. Physical and mental state had a minimal impact on the skin barrier.

The results of the skin melanin index were as follows: past medical history (37.97%) > skin allergy (18.25%) > living habits (16.71%) > cosmetic usage (13.79%) > physical and mental health (13.29%). The results of the skin erythema index were as follows: past medical history (53.79%) > living habits (18.79%) > skin allergies (13.42%) > cosmetic usage (7.16%) > physical and mental health (6.84%). The results of skin yellowness (b*) were as follows: past medical history (27.96%) > living habits (23.75%) > skin allergies (21.94%) > cosmetic usage (17.42%) > physical and mental health (8.92%). The results of the skin ITA° were as follows: skin allergy (32.57%) > living habits (24.02%) > past medical history (19.49%) > cosmetic usage (14.21%) > physical and mental health (9.71%) (Figure 5). Overall, physical and mental health had the smallest impact on skin tone, followed by cosmetic usage. Skin allergy had the largest impact on the ITA°, and past medical history had the largest impacts on the other indicators of skin tone (melanin, erythema, b*).

## 3 Supplementary tables

Table S1 62 survey questions and mapping IDs

| **Survey question** | **ID** |
| --- | --- |
| Which of the following is your work environment (or where you are often active)? | A1 |
| Your work and rest situation? | A2 |
| Have you ever smoke? | A3 |
| Have you ever drink？ | A4 |
| Are you have drug dependence？ | A5 |
| When you sleep regularly？ | A6 |
| Please evaluate your daily sleep duration. | A7 |
| Please evaluate your daily sleep quality. | A8 |
| Please evaluate your daily bowel movements. | A9 |
| The amount days of your menstrual period. | A10 |
| Please evaluate your menstrual blood volume when during menstrual period. | A11 |
| the amount days between your each menstrual periods. | A12 |
| Are you irritable or impulsive？ | A13 |
| Do you have insomnia and dreams？ | A14 |
| Do you feel that you have enough energy (mental and physical) in everday? | A15 |
| Are you stressed? | A16 |
| Is your daily diet greasy？ | A17 |
| Is your daily diet sweet？ | A18 |
| Is your daily diet salty？ | A19 |
| Is your daily diet spicy？ | A20 |
| Do you have the habit of exercising? | A21 |
| Do you ever have gastritis? | A22 |
| Do you ever have anemia? | A23 |
| Do you ever have bronchial asthma? | A24 |
| Do you ever have bronchitis? | A25 |
| Do you ever have hypothyroidism? | A26 |
| Do you ever have hepatitis (chronic hepatitis B)? | A27 |
| Do you ever have enteritis? | A28 |
| Do you ever have pneumonia? | A29 |
| Do you ever have angina? | A30 |
| Do you ever have peptic ulcer? | A31 |
| Do you ever have nephritis? | A32 |
| Do you ever have high blood pressure? | A33 |
| Do you ever have hyperthyroidism? | A34 |
| Do you ever have a tumor? | A35 |
| Do you ever have diabetes? | A36 |
| Do you ever have rheumatoid arthritis? | A37 |
| Do you ever have other diseases? | A38 |
| Do you ever have skin allergies? | A39 |
| Do you ever have skin allergies to external environments such as sunlight, wind, and cold? | A40 |
| Do you ever have skin allergies to cosmetics? | A41 |
| Do you ever have skin allergy to detergents? | A42 |
| Do you ever have skin allergies to medicines? | A43 |
| Do you ever have a skin allergy to food? | A44 |
| Do you ever have skin allergies to contact substances such as clothing? | A45 |
| Do you ever have skin allergies to body wash, hand sanitizer, etc.? | A46 |
| Do you ever have eczema? | A47 |
| Do you ever have photodermatitis? | A48 |
| Do you ever have psoriasis? | A49 |
| Do you ever have contact dermatitis? | A50 |
| Do you ever have suffered from other skin diseases? | A51 |
| Do you use eye cream in daily skincare? | A52 |
| Do you use serum in daily skincare? | A53 |
| Do you use toner in daily skincare? | A54 |
| Do you use lotion in daily skincare? | A55 |
| Do you use a cleanser in daily skincare? | A56 |
| Do you use face cream in daily skincare? | A57 |
| Do you use essential oils in daily skincare? | A58 |
| Do you use gel in daily skincare? | A59 |
| Do you use color makeup products in daily skincare? | A60 |
| How often do you use skin care products? | A61 |
| How often do you use sunscreen? | A62 |

Table S2 Principal components related to each skin parameter

| **Skin parameters** | **Skin-related principal components** |
| --- | --- |
| pH | PA3、PA4、PA7、PA11 |
| MEXA | PA1、PA3、PA4、PA9、PA10、PA13 |
| ERYTH | PA2、PA4、PA6、PA8、PA9、PA11、PA13、PA14 |
| CM | PA3、PA4、PA7、PA11、PA13 |
| TEWL | PA1、PA6、PA10 |
| b* | PA1、PA3、PA4、PA8、PA10、PA11、PA13、PA14 |
| ITA° | PA1、PA5、PA6、PA8、PA10 |
| SM | PA1、PA3、PA4、PA8、PA9、PA10、PA11、PA14 |

## 3 Supplementary figures


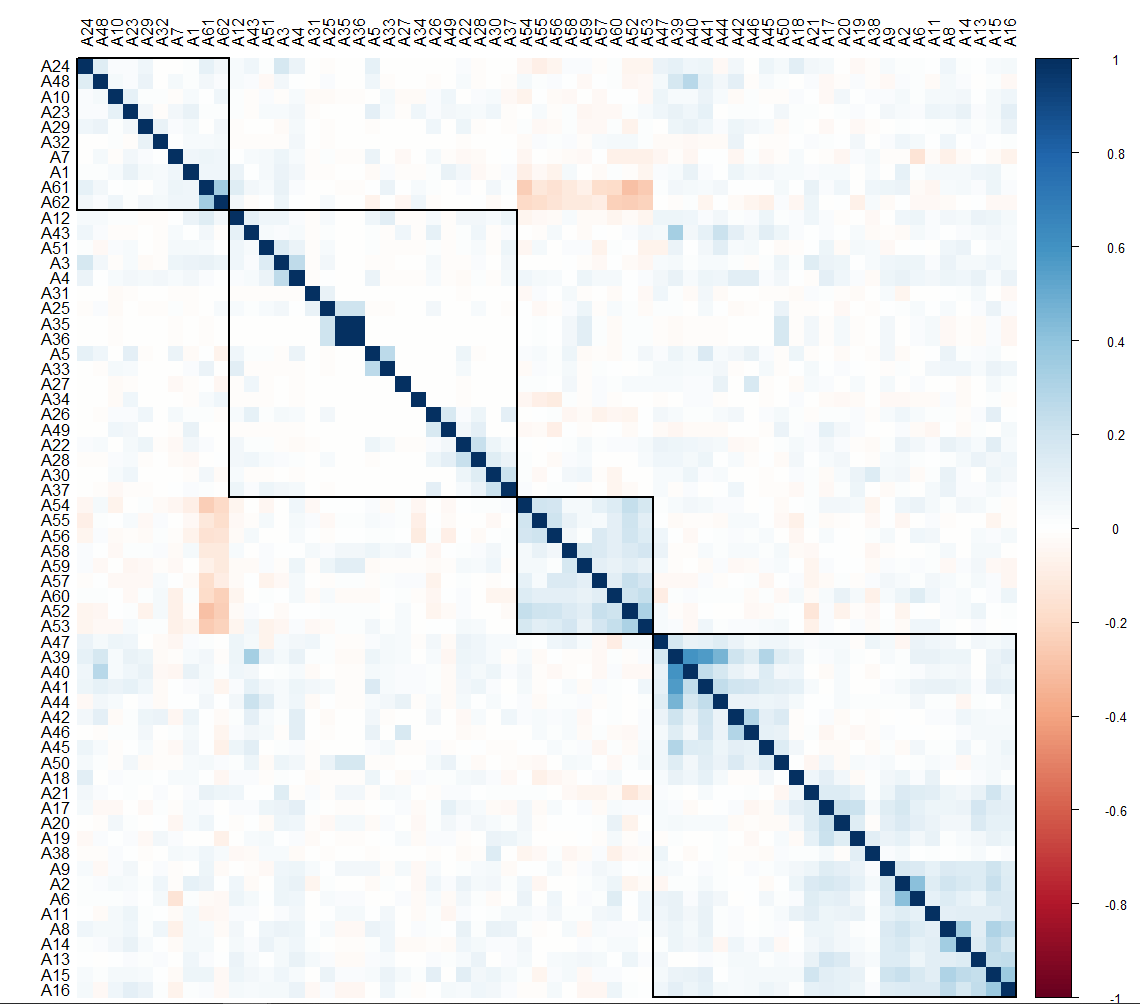


Figure S1 Cluster analysis of 62 questions


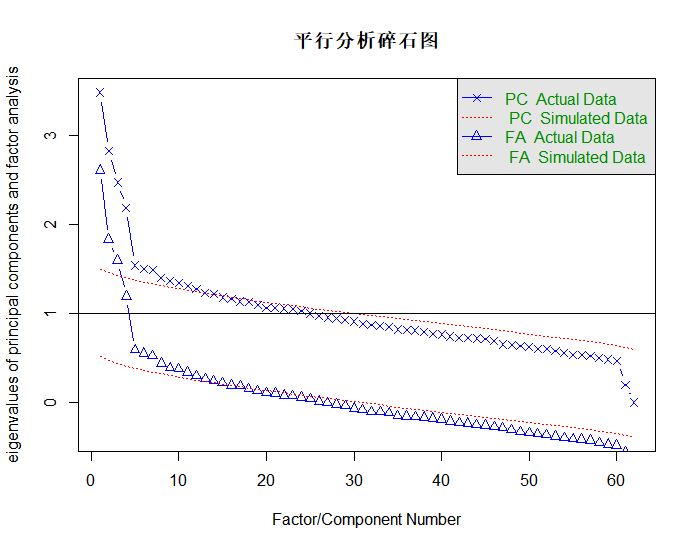


Figure S2 Gravel diagram of parallel analysis of 62 survey questions


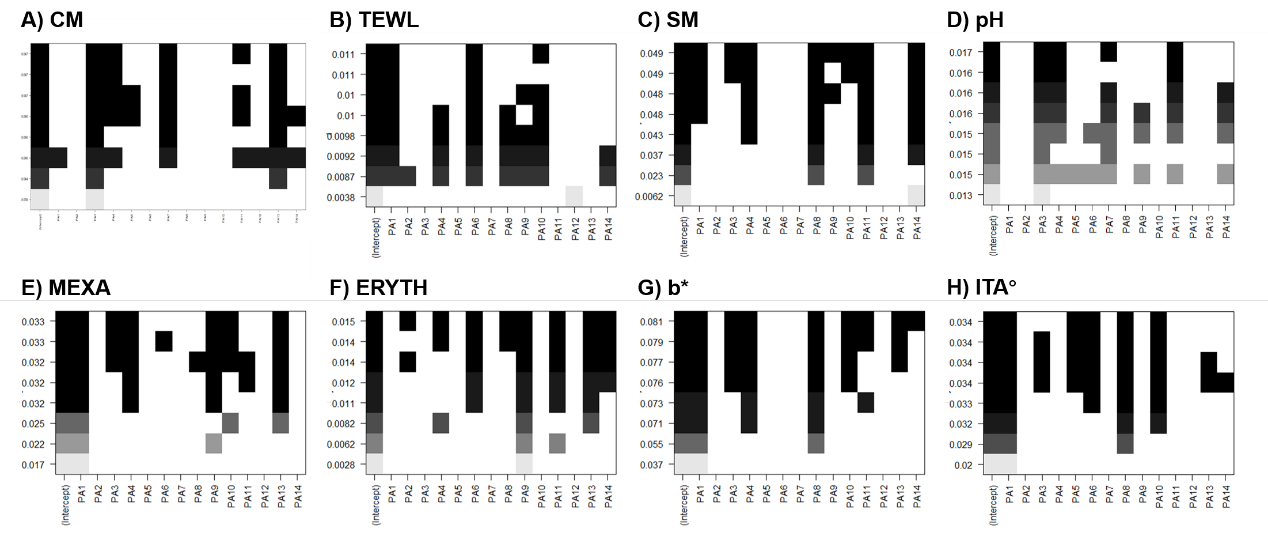


Figure S3 Optimal subset regression model of 8 skin parameters
